# Mass mortality in freshwater mussels (*Actinonaias pectorosa*) in the Clinch River, USA, linked to a novel densovirus

**DOI:** 10.1038/s41598-020-71459-z

**Published:** 2020-09-02

**Authors:** Jordan C. Richard, Eric Leis, Christopher D. Dunn, Rose Agbalog, Diane Waller, Susan Knowles, Joel Putnam, Tony L. Goldberg

**Affiliations:** 1 U.S. Fish and Wildlife Service, Southwestern Virginia Field Office, 330 Cummings Street, Abingdon, VA 24210 USA; 2 La Crosse Fish Health Center, Midwest Fisheries Center, U.S. Fish and Wildlife Service, 555 Lester Ave, Onalaska, WI 54650 USA; 3 Department of Pathobiological Sciences and Freshwater and Marine Sciences Program, University of Wisconsin-Madison, 1656 Linden Drive, Madison, WI 53706 USA; 4 U.S. Geological Survey, Upper Midwest Environmental Sciences Center, 2630 Fanta Reed Rd, La Crosse, WI 54603 USA; 5 U.S. Geological Survey, National Wildlife Health Center, 6006 Schroeder Rd, Madison, WI 53711 USA; 6 Global Health Institute, University of Wisconsin-Madison, 1300 University Avenue, Madison, WI 53706 USA

**Keywords:** Freshwater ecology, Infectious diseases, Limnology, Pathogens, Virology

## Abstract

Freshwater mussels (order Unionida) are among the world’s most biodiverse but imperiled taxa. Recent unionid mass mortality events around the world threaten ecosystem services such as water filtration, nutrient cycling, habitat stabilization, and food web enhancement, but causes have remained elusive. To examine potential infectious causes of these declines, we studied mussels in Clinch River, Virginia and Tennessee, USA, where the endemic and once-predominant pheasantshell (*Actinonaias pectorosa*) has suffered precipitous declines since approximately 2016. Using metagenomics, we identified 17 novel viruses in Clinch River pheasantshells. However, only one virus, a novel densovirus (*Parvoviridae*; *Densovirinae*), was epidemiologically linked to morbidity. Clinch densovirus 1 was 11.2 times more likely to be found in cases (moribund mussels) than controls (apparently healthy mussels from the same or matched sites), and cases had 2.7 (log_10_) times higher viral loads than controls. Densoviruses cause lethal epidemic disease in invertebrates, including shrimp, cockroaches, crickets, moths, crayfish, and sea stars. Viral infection warrants consideration as a factor in unionid mass mortality events either as a direct cause, an indirect consequence of physiological compromise, or a factor interacting with other biological and ecological stressors to precipitate mortality.

## Introduction

Freshwater mussels (order Unionida) are important members of freshwater biomes, providing ecosystem services such as water filtration, nutrient cycling and deposition, physical habitat stabilization, and food web enhancement^1^. Mussels filter-feed on bacteria, suspended algae, detritus, phytoplankton and zooplankton^2^, removing suspended particulate matter from the water column and from interstitial spaces within the substrate. During periods of low summer discharge in small rivers, mussel assemblages are capable of circulating water as it flows over them, leading to multiple cycles of filtration^3^ that can strongly influence ecosystem processes, even at moderate mussel densities^4^. Unionids are also gaining attention for their ability to filter out chemical contaminants and water-borne pathogens^5–7^.

Unfortunately, the order Unionida contains an exceptional number of imperiled taxa. Among North America’s 298 recognized unionid species^8^, > 70% are considered endangered, threatened, or vulnerable^9^, with 23 species having gone extinct from the Southeastern United States alone. Historically, habitat destruction (e.g., river impoundments), pollution, sedimentation, over-harvest for commercial use (most notably, pearl harvest and manufacture of shirt buttons from shells ca. 1850–1950)^10^, and competition from invasive species (e.g. the Asian clam *Corbicula fluminea,* zebra mussel *Dreissena polymorpha,* and quagga mussel *D. bugensis*)^11^ have greatly reduced or extirpated many native mussel fauna. These threats have been present since the early twentieth century, mirroring trends in human development and land use^12^.

Since the late 1970s, episodic mass mortality events have been documented in unionids throughout their range, including catastrophic mortality (> 90% population declines) in some cases^12^. Unlike the aforementioned gradual declines, many mass mortality events in freshwater mussels have not been directly attributed to any specific environmental changes or events^12^. Furthermore, mass mortality events often affect only a single species of mussel within a broad ecological community. Environmental factors (e.g. chemical spills, extreme weather events) would be expected to affect many or all unionid species, in addition to other invertebrates and fishes^13^. A meta-analysis of the causes of mussel population declines found that only 48% of studies could attribute declines to any particular cause, and over 75% of studies cited multiple causes without substantial evidence of mechanisms^14^.

The Clinch River watershed in southwestern Virginia and northeastern Tennessee is one of the most ecologically important and biodiverse freshwater systems in North America^15^. With 46 extant species of freshwater mussels (20 of which are federally listed as endangered) and over 100 species of fish (5 of which are federally listed as either threatened or endangered), the Clinch River supports the highest concentration of extant federally listed aquatic species in the USA^16^. Long-term quantitative monitoring has shown that mussel richness and abundance in the upper river in Virginia steadily fell from 1979 to 2014, with densities at some sites declining as much as 95%^16^. In contrast, mussel densities in the lower river in Tennessee increased from 1979 to 2014^17^. Several studies have examined Clinch River water and sediment quality and their effects on freshwater mussel assemblage in an attempt to explain this “zone of decline,” but few direct links to water quality, sediment, or physical habitat quality have been identified^18^.

Beginning in summer 2016, field biologists began documenting mass die-offs of mussels within the “healthy” reach of the lower Clinch River^19^. Mortality episodes were characterized by large numbers of recently dead or dying mussels on the surface of the river substrate in late summer and fall. Field surveys, collection of shells from freshly dead mussels, and comparisons to known species assemblage patterns demonstrated that the pheasantshell (*Actinonaias pectorosa*) comprised a disproportionate (to their relative abundance within the community) and overwhelming majority of affected individuals^17^. These mortality events resulted in population declines of approximately 50–90% of pheasantshells at monitoring sites throughout the lower river. For example, at one monitoring site (Kyle’s Ford), data from yearly quantitative surveys documented a loss of 85.4% of the pheasantshell population from 2016 to 2019, translating to a loss of approximately 80,000 individuals from this 200-m reach of the Clinch River^19^. Remarkably, similar mass mortality has not been observed in the other species of mussels inhabiting the same areas of the river. Moreover, since 2016, mass mortality of pheasantshells has occurred in upstream sites originally considered unaffected^19^. Pheasantshell are large-bodied and abundant, historically comprising over 50% of the Clinch River’s mussel biomass^16^. Thus, there is great concern that this decline, if unchecked, could permanently alter the Clinch River’s ecology and irreversibly affect the ecosystem services that its mussels provide.

Here, we describe a multi-year investigation into the Clinch River pheasantshell die-off focusing on infection, which has been cited as a potential—even likely—cause for unionid die-offs^13,20,21^ but has remained understudied^22^. This study is part of a broader collaborative effort to investigate potential causes for pheasantshell die-offs in the Clinch River and elsewhere^23^. We focus on viral causes because of (1) the specificity of the die-off for pheasantshells, (2) the apparent upstream spread of pheasantshell mortality between 2016 and 2019, (3) lack of evidence for bacterial or eukaryotic etiological agents^24,25^, and (4) lack of evidence of changes in physical characteristics of the environment that might explain the die-off^17^. Moreover, viruses are known to cause epidemic mortality in marine bivalves^26,27^, and Lea plague virus can decimate farmed populations of Chinese triangleshell (*Hyriopsis cumingii*) freshwater mussels used for production of freshwater pearls^28,29^. We took advantage of advances in metagenomic technologies for detecting and characterizing unknown viruses and viral communities, which have proven useful for elucidating the invertebrate “virosphere”^30,31^. By applying these methodologies alongside a rigorous case–control study design in which we compared affected and unaffected animals during two consecutive years (2017 and 2018), were able to examine which constituents of the pheasantshell virome might be associated with disease.

## Results

### Sampling

**Figure 1:**
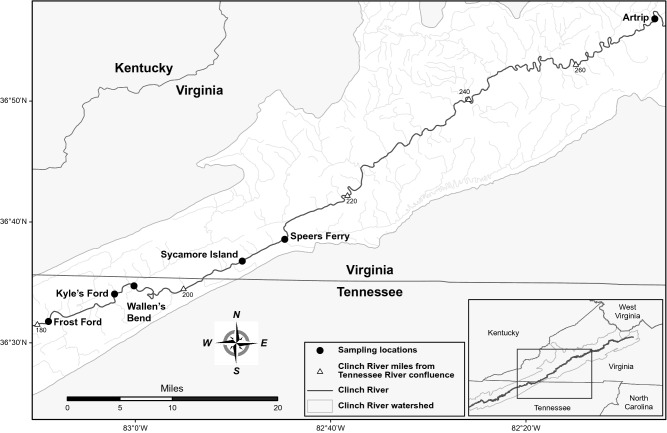
Map of sampling locations. The map was created using ArcMap version 10.4.1 (Esri, Redlands, California, USA; https://support.esri.com/en/products/desktop/arcgis-desktop/arcmap/10-4-1).

We collected and analyzed samples from 58 pheasantshells from the Clinch River, including 26 cases (11 from 2017 and 15 from 2018) and 32 controls (8 from 2017 and 24 from 2018) at 6 sites (Fig. 1; Table S1). During sampling, we chose as cases mussels that were on the surface of the substrate, gaping, slow to respond to tactile stimuli, and able to close their valves only weakly, and we chose as controls mussels that were firmly buried in the substrate, fast to respond to tactile stimuli, and able to close their valves strongly. In 2017, we sampled in October and November 2017 during an active mass mortality event. In 2018, we began sampling in August, before mortality was observed, and we continued sampling during September and October when mass mortality did occur. Prolonged flood conditions immediately after the October 2018 sampling event prevented further sample collection in 2018.

### Viromics and statistical analyses

**Figure 2:**
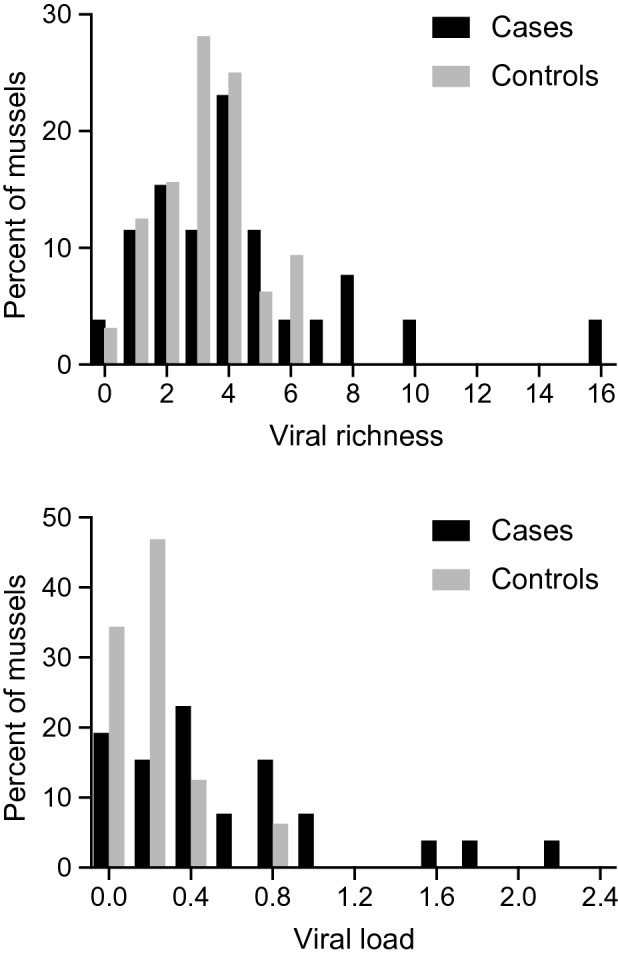
Frequency distribution of viral richness (number of viruses) and viral load (log_10_ viral reads per 10^6^ total reads per kilobase of target sequence) in Clinch River pheasantshell cases and controls.

Metagenomic sequencing of 58 pheasantshells from the Clinch River yielded an average of 1,921,287.6 sequence reads per hemolymph sample (standard deviation 1,127,991.5) with an average length of 118.3 nucleotides (standard deviation 10.6), after length and quality trimming. De novo assembly of these reads yielded 20,058 contiguous sequences (contigs) averaging 1,671 nucleotides in length (range 856–92,913). From these data, we identified 17 viruses of varied genomic compositions and taxonomic classifications (Table 1). Most viruses are only distantly related to known viruses phylogenetically, but many are related to viruses of freshwater and marine mollusks and other invertebrates (Fig. S1). Mussels identified as cases harbored an average of 4.4 (standard error = 0.66) viruses, whereas mussels identified as controls harbored an average of 3.2 (SE = 0.27) viruses, and this difference was statistically significant (*t* = 1.839; df = 56; *P* = 0.0356). Average loads of all viruses were 0.135 log_10_ viral reads per million total reads per kilobase of target sequence (vRPM/kb) for cases and 0.064 log_10_ vRPM/kb for controls, and this difference was also statistically significant (*t* = 3.706; df = 54; *P* = 0.0003). Frequency distributions of viral richness and viral load were right-skewed for cases but less so for controls, with some cases having exceptionally high viral richness and viral loads (Fig. 2).

**Table 1:** Viruses identified in Clinch River pheasantshells.

ID^1^	Virus name	Accession	Genome	Closest relative (source, location, year, accession)^2^	Family^3^	Genus^3^	%ID (aa)^2^
A	Clinch densovirus 1	MT341473	ssDNA (linear)	Periplaneta fuliginosa densovirus (cockroach, China, 1990, AF192260)	*Parvoviridae*	*Ambidensovirus*	63.7
B	Clinch narna-like virus 1	MT341474	ssRNA(+)	Sanxia narna-like virus 2 (shrimp, China, 2014, KX883567)	Unclassified	Unclassified	45.4
C	Clinch noda-like virus 1	MT341475	ssRNA(+)	Hubei noda-like virus 2 (freshwater shellfish, China, 2014, KX883205)	Unclassified	Unclassified	51.9
D	Clinch picorna-like virus 1	MT341476	ssRNA(+)	Marine RNA virus SF-2 (wastewater, USA, 2010, NC_043518)	*Marnaviridae*	*Locarnavirus*	41.9
E	Clinch CRESS virus 1	MT341477	ssDNA (circular)	CRESS virus (minnow, USA, 2017, MH616916)	Unclassified	Unclassified	61.7
F	Clinch picorna-like virus 2	MT341478	ssRNA(+)	Hubei picorna-like virus 4 (freshwater shellfish, China, 2014, NC_033087)	Unclassified	Unclassified	65.8
G	Clinch picorna-like virus 3	MT341479	ssRNA(+)	Wenzhou picorna-like virus 7 (shrimp, China, 2013, NC_032842)	Unclassified	Unclassified	55.7
H	Clinch circular virus 1	MT341480	ssDNA (circular)	Blackfly DNA virus 6 (black flies, New Zealand, 2015, MK433220)	Unclassified	Unclassified	70.1
I	Clinch calicivirus 1	MT341481	ssRNA(+)	Bat calicivirus (bat, USA, 2009, MH259583)	*Caliciviridae*	*Calicivirus*	80.2
J	Clinch circular virus 2	MT341482	ssDNA (circular)	Bat circovirus (bat, China, 2013, KJ641738)	*Circoviridae*	Unclassified	97.5
K	Clinch dicistro-like virus 1	MT341483	ssRNA(+)	Beihai picorna-like virus 105 (snails, China, 2014, NC_032604)	Unclassified	Unclassified	79.1
L	Clinch tombus-like virus 1	MT341484	ssRNA(+)	Hubei tombus-like virus 15 (centipede, China, 2013, NC_033009)	*Tombusviridae*	Unclassified	63.8
M	Clinch sobemo-like virus 1	MT341485	ssRNA(+)	Beihai sobemo-like virus 25 (razor shell, China, 2014, NC_032895)	*Luteoviridae*	Unclassified	65.6
N	Clinch dicistro-like virus 2	MT341486	ssRNA(+)	Hypsignathus monstrosus dicistrovirus (bat, Republic of the Congo, 2015, MH310078)	*Dicistroviridae*	Unclassified	63.0
O	Clinch picobirnavirus 1	MT341487	dsRNA (segmented)	Pink-eared duck picobirnavirus (duck, Australia, 2017, MK204418)	*Picobirnaviridae*	*Picobirnavirus*	64.1
P	Clinch picobirna-like virus 1	MT341488	ssRNA(+)	Shahe picobirna-like virus 1 (freshwater isoptera, China, 2013, KX884156)	Unclassified	Unclassified	76.5
Q	Clinch totivirus 1	MT341489	ssRNA(+)	Drosophila melanogaster totivirus (fruit fly, USA, 2009, NC_013499)	*Totiviridae*	Unclassified	96.0

^1^Letters refer to Table 2, Figs. 3, and S1.

^2^Closest phylogenetic relative in the GenBank database; see Fig. S1.

^3^Family, genus and percent amino acid identity to the closest phylogenetic relative in the GenBank database.

**Figure 3:**
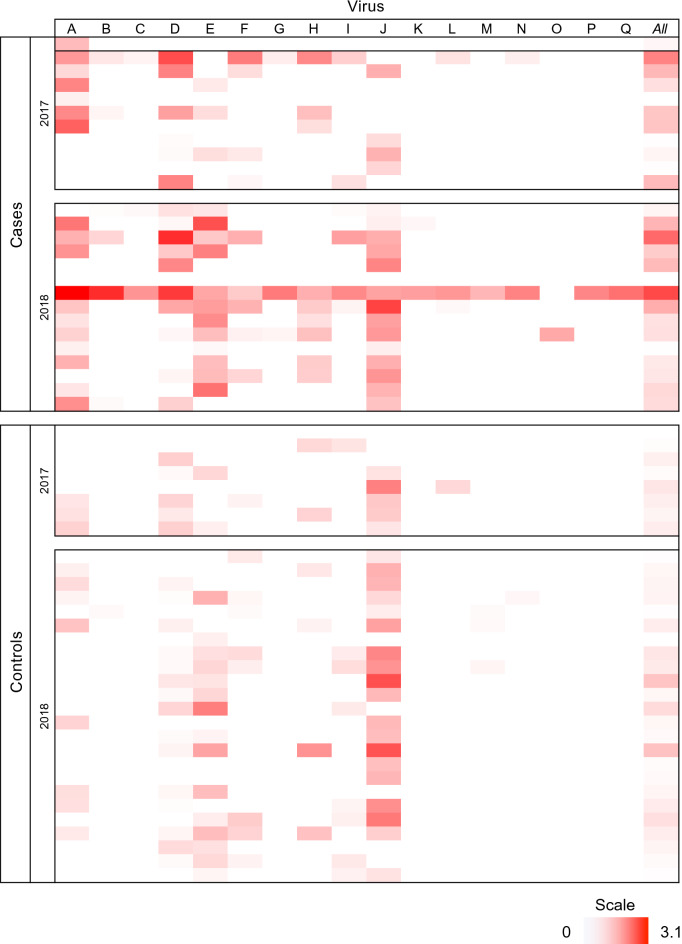
Heatmap of viral loads in Clinch River pheasantshells. Data are log_10_ viral reads per 10^6^ total reads per kilobase of target sequence for each virus separately (viruses A–Q) and for all viruses combined (*All*). Data are presented separately for cases and controls in 2017 and 2018. Raw data on viral loads are presented in Table S2.

Individual viruses varied markedly in their prevalence, load, and association with case or control status (Table 2). In univariate analyses, five viruses (Clinch densovirus 1, Clinch narna-like virus 1, Clinch noda-like virus 1, Clinch picorna-like virus 1, and Clinch CRESS virus 1) showed significantly higher prevalence and/or viral load in cases than in controls (Table 2). Two of these viruses were relatively rare: Clinch narna-like virus 1 was found in 6 cases and 1 control and Clinch noda-like virus 1 was found in 3 cases and 0 controls. Two other of these viruses (Clinch picorna-like virus 1 and Clinch CRESS virus 1) had higher viral loads in cases than in controls but showed no significant differences in prevalence between cases and controls. Thus, Clinch densovirus 1 was the only virus for which both prevalence and load were significantly higher in cases than in controls (odds ratio (OR) = 4.30, 95% confidence interval (CI) 1.42–13.0; *P* = 0.0084, and Mann–Whitney U = 40, *P* = 0.0035, respectively). The remaining 12 viruses showed no statistically significant differences in prevalence or load between cases and controls overall or within years (Table 2; Fig. 3).

**Table 2:** Univariate statistical associations between clinical classification (case or control) and prevalence and loads of viruses in Clinch River pheasantshells.

ID^1^	Virus name	Individuals infected	Prevalence (%)^2^				Viral load (Log_10_vRPM/kb)^3^			
			Cases	Controls	OR (95% CI)	*P*	Cases	Controls	U	*P*
A	Clinch densovirus 1	29	69.2	34.4	4.30 (1.42, 13)	**0.0084**	1.057	0.396	40	**0.0035**
B	Clinch narna-like virus 1	7	23.1	3.1	9.30 (1.041, 83.12)	**0.0267**	0.601	0.074	n/a	n/a
C	Clinch noda-like virus 1	3	11.5	0.0	9.68 (0.4771, 196.4)	**0.0360**	0.512	0.000	n/a	n/a
D	Clinch picorna-like virus 1	36	61.5	62.5	0.96 (0.3306, 2.788)	0.9999	0.992	0.225	78	**0.00415**
E	Clinch CRESS virus 1	32	57.7	53.1	1.20 (0.4241, 3.413)	0.4676	0.911	0.544	80	**0.03785**
F	Clinch picorna-like virus 2	18	34.6	28.1	1.35 (0.443, 4.132)	0.4017	0.628	0.289	21	0.0939
G	Clinch picorna-like virus 3	3	11.5	0.0	9.68 (0.4771, 196.4)	0.0843	0.657	0.000	n/a	n/a
H	Clinch circular virus 1	15	34.6	18.8	2.29 (0.6908, 7.619)	0.1423	0.737	0.592	17	0.13605
I	Clinch calicivirus 1	14	23.1	25.0	0.90 (0.2675, 3.028)	0.9999	0.630	0.254	6	0.1725
J	Clinch circular virus 2	42	69.2	75.0	0.75 (0.2363, 2.38)	0.8435	0.930	0.946	207	0.8308
K	Clinch dicistro-like virus 1	2	7.7	0.0	6.63 (0.3045, 144.5)	0.1966	0.638	0.000	n/a	n/a
L	Clinch tombus-like virus 1	4	11.5	3.1	4.04 (0.3948, 41.41)	0.2314	0.567	0.471	n/a	n/a
M	Clinch sobemo-like virus 1	4	3.8	9.4	0.39 (0.03779, 3.956)	0.7774	0.895	0.092	n/a	n/a
N	Clinch dicistro-like virus 2	3	7.7	3.1	2.58 (0.221, 30.2)	0.4213	0.859	0.101	n/a	n/a
O	Clinch picobirnavirus 1	1	3.8	0.0	3.82 (0.1494, 97.84)	0.4483	1.048	0.000	n/a	n/a
P	Clinch picobirna-like virus 1	1	3.8	0.0	3.82 (0.1494, 97.84)	0.4483	1.473	0.000	n/a	n/a
Q	Clinch totivirus 1	1	3.8	0.0	3.82 (0.1494, 97.84)	0.4483	1.727	0.000	n/a	n/a

^1^Letters refer to Table 1, Figs. 3, and S1.

^2^Percentage of mussels within each group (case or control) with reads mapping to each virus, plus odds ratios and 95% confidence intervals. *P* values (statistically significant values in bold) were calculated using Fisher's exact tests.

^3^Log_10_ reads mapping to each virus per million total reads per kilobase of target sequence, Mann–Whitney U statistics, and associated *P* values (infected mussels only).

Based on the results described above, we conducted multivariate statistical analyses that included five viruses (Clinch densovirus 1, Clinch narna-like virus 1, Clinch noda-like virus 1, Clinch picorna-like virus 1, and Clinch CRESS virus 1) because of their significantly higher prevalence and/or load in cases than in controls. In the resulting general linear model (GLM) relating clinical status to viral infection and ecological variables, only two significant factors emerged: infection with Clinch densovirus 1 [*P* = 0.004, adjusted OR (95% CI) = 11.18 (2.12–58.92)] and mussel shell length [*P* = 0.043, adjusted OR (95% CI) = 1.09 (1.00–1.17)]. In the GLM relating clinical status to viral load and ecological variables, the only significant factor identified was Clinch densovirus 1 load [*P* = 0.0287, adjusted OR (95% CI) = 24.56 (1.39, 432.52)]; no other viruses and no ecological factors were significant. The general linear model relating viral richness to ecological factors (site, sampling date, and length) had no significant terms.

Because of the strong associations of Clinch densovirus 1 prevalence and load with morbidity, we examined associations between Clinch densovirus 1 and the presence and load of other viruses using Fisher's exact tests and Student’s *t* tests, respectively. Infection with Clinch densovirus 1 was associated with a higher frequency of infection with Clinch circular virus 1 (odds ratio = 5.9 [95% CI 1.33–37.6] Fisher's 1-tailed exact *P* = 0.007) and with a higher load of Clinch CRESS virus 1 (t = 2.527; df = 26.185; *P* = 0.0179); however, no other significant associations were detected.

The genome of Clinch densovirus 1 (GenBank accession number MT341473) is 5,429 bases long and contains 5 open reading frames (ORFs 1–5) of lengths 735, 1,671, 1,620, 807, and 759 nucleotides in the typical arrangement of members of subfamily *Densovirinae*, encoding putative non-structural and structural proteins, which are transcribed by host cellular machinery through alternative mRNA splicing and leaky scanning^32,33^. The Clinch densovirus 1 coding genome is also flanked by inverted terminal repeats characteristic of members of this viral subfamily^32^. The amino acid sequence difference between Clinch densovirus 1 and its closest relative, periplaneta fuliginosa densovirus, a member of the genus *Ambidensivirus* (Table 1), is 63.7% within the non-structural protein NS1. This degree of divergence exceeds the 85% relatedness threshold accepted by the International Committee on the Taxonomy of Viruses as a species demarcation criterion within the genus *Ambidensovirus*^34^.

## Discussion

Clinch River pheasantshells host a diverse virome. Three of the 17 viruses we identified (Clinch picorna-like virus 1, Clinch CRESS virus 1, and Clinch circular virus 2; Table 1) are likely members of the “normal” pheasantshell virome. Such viruses would be expected to infect mussels at high prevalence (> 50% in these cases) and load, but without association with clinical disease. Three other viruses infected pheasantshells at moderate prevalence (between 20 and 50%) but also showed no association between case and control status (Clinch picorna-like virus 2, Clinch circular virus 1, and Clinch calicivirus 1). The other viruses we identified all occurred at low prevalence (sometimes in only one animal) and may be hypoendemic, sporadic, or derived from the environment. For example, the picobirnavirus detected in one case sample from 2018 is part of a group of viruses shed in the feces of mammals such as cows and marmots^35^. Although hemolymph, like mammalian blood, is not directly connected to the environment^36^, filter feeding bivalves can remove viral pathogens from suspension in the water column^37,38^.

Among the five viruses with prevalence or loads associated with case status by univariate analyses (Table 1), only Clinch densovirus 1 had both higher prevalence and load in cases than in controls, and these associations were the strongest observed in the study. In multivariate analyses, the other four viruses fell out as non-significant with respect to both prevalence and load, as did all other factors except for mussel shell length, which was retained in the GLM examining prevalence. Clinch densovirus 1 is therefore the only of the 17 viruses identified that, when other variables are accounted for, is associated with disease in Clinch River pheasantshells.

Densoviruses are members of the viral family *Parvoviridae*, subfamily *Densovirinae*, and can be highly host-specific and lethal^39^. Mass mortality in invertebrates is a well-characterized consequence of densovirus infection*,* with examples including shrimp^40^, silkworms^41^, cockroaches^42^, mosquitos^43^, crickets^44^, moths^45^, and crayfish^46^. In fact, so lethal are some densoviruses that they have been used commercially as powerful bioinsecticides^47^. Common signs of densovirus infection include lethargy, anorexia, development of tumors, flaccidity, and death^39^. Notably, sea star-associated densovirus, also a member of the genus *Ambidensovirus*, is the putative cause of mass mortality in another benthic invertebrate, the sunflower sea star (*Pycnopodia helianthoides*)^48^. Sea star wasting disease is characterized by loss of turgor (a “deflated” appearance), behavioral changes, and rapid degradation leading to death^48^.

Henley et al.^24^ conducted a histological study of moribund Clinch River pheasantshells collected during the beginning of the die-off in 2016 from the Kyle’s Ford sampling site. This study documented internal organ damage, including pervasive necrosis, but was unable to link any measured factor (including parasitic trematode infestation and bacterial infection), to mortality. Certain of the histologic lesions documented, however, would be consistent with densovirus infection, as described in other invertebrates (see above). Ultimately, experimental infection and studies of pathogenesis will be necessary to resolve any causal relationship between phesantshell mussel mortality and infection with Clinch densovirus 1, as has been attempted in the case of sea star wasting disease^48^ and cherax quadricarinatus densovirus^46^.

In this light, we caution that our results, while suggestive, do not demonstrate direction of causality. For example, a preceding diseased state may render mussels more susceptible to infection with Clinch densovirus 1. We also note that we characterized viruses from hemolymph, because it is useful for bivalve health assessment and can be obtained non-lethally^36,49^, but other tissues may host different viruses. Our focus on hemolymph may also account for our finding of only relatively small viruses (similar to vertebrate blood). Other (and perhaps larger) viruses may have tropisms for different tissues (e.g. mantle, gill, gonads), and these tissues also warrant investigation. Quantitative polymerase chain reaction assays could be developed to measure the tissue-specific loads of viruses determined by epidemiology and metagenomics to be linked to disease states, including Clinch densovirus 1 but not dismissing other viruses (discovered and as-yet undiscovered). Such assays could also be applied to environmental samples (e.g. water or sediment) to investigate viral transmission and persistence.

Should infection with Clinch densovirus 1 or other pathogens ultimately be a cause of pheasantshell mass mortality, this result would not exclude the possibility of “upstream drivers.” Infectious diseases are often proximate causes of mortality while also being caused by other factors themselves. For example, introductions of exotic species and their pathogens, climate change, and ecologically induced physiological stressors have all been implicated as predisposing factors for infectious disease in wildlife^50^. Determining proximate causes is nevertheless important for management and conservation. For example, vaccines, probiotics, or controlled exposure to pathogens to induce resistance might be effective in conditioning mussels in captive rearing facilities, where many species are bred for restoration efforts^51^.

Overall, our results show that, while diverse, the virome of Clinch River pheasantshells contains only one virus, Clinch densovirus 1, showing a strong and consistent association with disease. Mass mortality events in freshwater mussels are unfortunately accelerating worldwide^12^. Studying other species of mussels in other geographic locations using both epidemiology and metagenomics could help reveal whether infection with viruses or other agents is a generalized characteristic of unionid mass mortality events. The resulting information would be important for conserving and managing this remarkable complex of imperiled species.

## Methods

### Field sampling

We sampled pheasantshells in 2017 and 2018. We collected moribund mussels (cases) and apparently healthy mussels (controls) during mortality events using swim-through searches of shoals. We focused on the months of September, October and November of each year because these were the months in which mass mortality was observed, although we added a sampling event in August 2018 in anticipation of a mortality event. At four sites along the river (Frost Ford, Kyle’s Ford, Wallen’s Bend, and Sycamore Island; Fig. 1), we first located moribund individuals (lying on the surface with shells gaping and minimally responsive to tactile stimuli). We then located apparently healthy individuals (buried in the substrate, siphoning normally, with tightly closed shells and strongly resistant to being opened) at the same sites and from two additional upstream sites (Speers Ferry and Artrip) where no mortality had been observed.

We sampled hemolymph because it is useful for health and disease assessment in freshwater bivalves and can be collected non-lethally^36,49^ and because (similar to vertebrate blood) it is not directly exposed to the physical environment, unlike other accessible organs (e.g. foot, mantle, gill). We first gently opened the valves of each animal with a sterile pediatric nasal speculum. We then disinfected the outer surface of the anterior adductor muscle with 70% isopropyl alcohol and extracted up to 1.0 ml hemolymph (depending on the size of the mussel) from the anterior adductor muscle sinus using a 1 ml tuberculin syringe. We placed hemolymph immediately in sterile tubes on dry ice in the field then stored samples at − 80 °C until further analysis. For each individual, we noted its general appearance, recorded the strength and speed of its response to tactile stimuli (opening the valves and application of isopropanol), and measured the length of its shell using digital calipers. We marked animals with FPN glue-on shellfish tags (Hallprint, Hindmarsh Valley, Australia) to prevent re-sampling during successive sampling events and then returned animals to the shoals from which they were collected.

### Metagenomic sequencing and bioinformatics

We processed hemolymph for metagenomic sequencing for virus discovery as described previously^52^. Briefly, we clarified hemolymph by centrifugation at 10,000×*g* for 10 min and used the QIAamp MinElute virus kit (Qiagen, Hilden, Germany) to extract total nucleic acids from the supernatant. We then converted RNA to double-stranded cDNA using random hexamers and prepared libraries using the Nextera XT DNA sample preparation kit (Illumina, San Diego, California, USA), after which we sequenced the libraries on an Illumina MiSeq instrument (V3 chemistry, 600 cycle kit; Illumina, San Diego, California, USA). Using CLC Genomics Workbench version 11.0 (CLC bio, Aarhus, Denmark), we first trimmed low-quality bases (phred quality score < 30) and discarded short reads (< 75 bp). We then conducted de novo assembly using the native CLC assembler with a word size of 50 and a bubble size of 5,000 and analyzed both contigs and unassembled reads for nucleotide-level (blastn) and protein-level (blastx) similarity to viruses in the GenBank database. For each mussel, we measured its infection status (positive or negative) for each virus and, for infected mussels, vRPM/kb, which is a metagenomic measure of viral load that adjusts for sequencing depth and target sequence length and is correlated with quantitative real-time PCR^52^.

We inferred phylogenetic relationships among newly identified virus sequences and published sequences of the most closely related viruses in the GenBank database using viral replicase (polymerase) genes when available. We first aligned sequences using a codon-based version of the Prank algorithm^53^ and applied the Gblocks algorithm^54^ to remove regions with poor alignment, as implemented in the computer program TranslatorX^55^. We then inferred maximum likelihood phylogenetic trees from the resulting alignments using PhyML 3.0^56^, with 1,000 bootstrap replicates to assess statistical confidence in clades. We used FigTree v1.4.4 to display final trees.

### Statistical analyses

We used a multi-tiered statistical approach to examine associations between viral infection, load, and richness (total number of viruses infecting a mussel) and clinical status (cases versus controls). First, we used Fisher’s exact tests and Mann–Whitney U tests to assess univariate statistical differences between cases and controls with respect to these measures. Based on the results of these univariate analyses (Table 2), we constructed general linear models to investigate the combined influence of viruses and other predictor variables (shell length, sampling location, and date of sampling) on clinical status (case or control). We conducted all statistical analyses using R software^57^.

### Ethics statement

We obtained biological samples in accordance with all federal, state, and local laws and policies.

## Supplementary information

Supplementary Information.

## Data Availability

All data generated during the current study are available in GenBank (accession numbers MT341473–MT341489) or are included in this published article and its Supplementary Information files.
