# Supplementary material for: Mass mortality in freshwater mussels (Actinonaias pectorosa) in the Clinch River, USA, linked to a novel densovirus
Source: Sci Rep. 2020 Sep 2;10:14498. doi: 10.1038/s41598-020-71459-z (PMC7468154; doi:10.1038/s41598-020-71459-z)
Supplement: Supplementary file 1 — Supplementary Information. [file 41598_2020_71459_MOESM1_ESM.pdf]

SUPPLEMENTARY INFORMATION[1]

Mass mortality in freshwater mussels (*Actinonaias pectorosa*) in the Clinch River, USA, linked to a novel densovirus

Jordan Richard[1,3], Eric Leis[2], Christopher D. Dunn[3], Rose Agbalog[1], Diane Waller[4], Susan Knowles[5], Joel Putnam[4], and Tony L. Goldberg[3,6]*

[1]U.S. Fish and Wildlife Service, Southwestern Virginia Field Office, 330 Cummings Street, Abingdon, VA 24210. [2]La Crosse Fish Health Center, Midwest Fisheries Center, U.S. Fish and Wildlife Service, 555 Lester Ave, Onalaska, WI, 54650. [3]Department of Pathobiological Sciences, University of Wisconsin-Madison, 1656 Linden Drive, Madison WI 53706. [4]U.S. Geological Survey, Upper Midwest Environmental Sciences Center, 2630 Fanta Reed Rd, La Crosse WI, 54603. [5]US Geological Survey, National Wildlife Health Center, 6006 Schroeder Rd, Madison, WI 53711. [6]Global Health Institute, University of Wisconsin-Madison, 1300 University Avenue, Madison WI 53706

*Correspondence and requests for materials should be addressed to Tony L. Goldberg, Department of Pathobiological Sciences, University of Wisconsin-Madison, 1656 Linden Drive, Madison, Wisconsin 53706, USA. Email: tony.goldberg@wisc.edu; Phone: +1-608-890-2618; Fax: +1-608-262-7420.

Table S1. Details of Clinch River pheasantshells (*Actinonaias pectorosa*) used in the analyses

Table S2. Loads of 17 viruses in Clinch River pheasantshells (*Actinonaias pectorosa*)

Figure S1. Maximum likelihood phylogenetic trees of 17 viruses identified in Clinch River pheasantshells (*Actinonaias pectorosa*) and their relatives in the GenBank database. Taxon names indicate host, country, year of collection, and GenBank accession number; see Table 2 for additional details. Viruses identified in the present study are shown in bold type. Numbers beside branches are bootstrap values representing statistical confidence in clades based on 1000 resamplings of the data; only bootstrap values ≥50% are shown. Scale bars indicate nucleotide substitutions per site.

[1]Any use of trade, firm, or product names is for descriptive purposes only and does not imply endorsement by the U.S. Government.

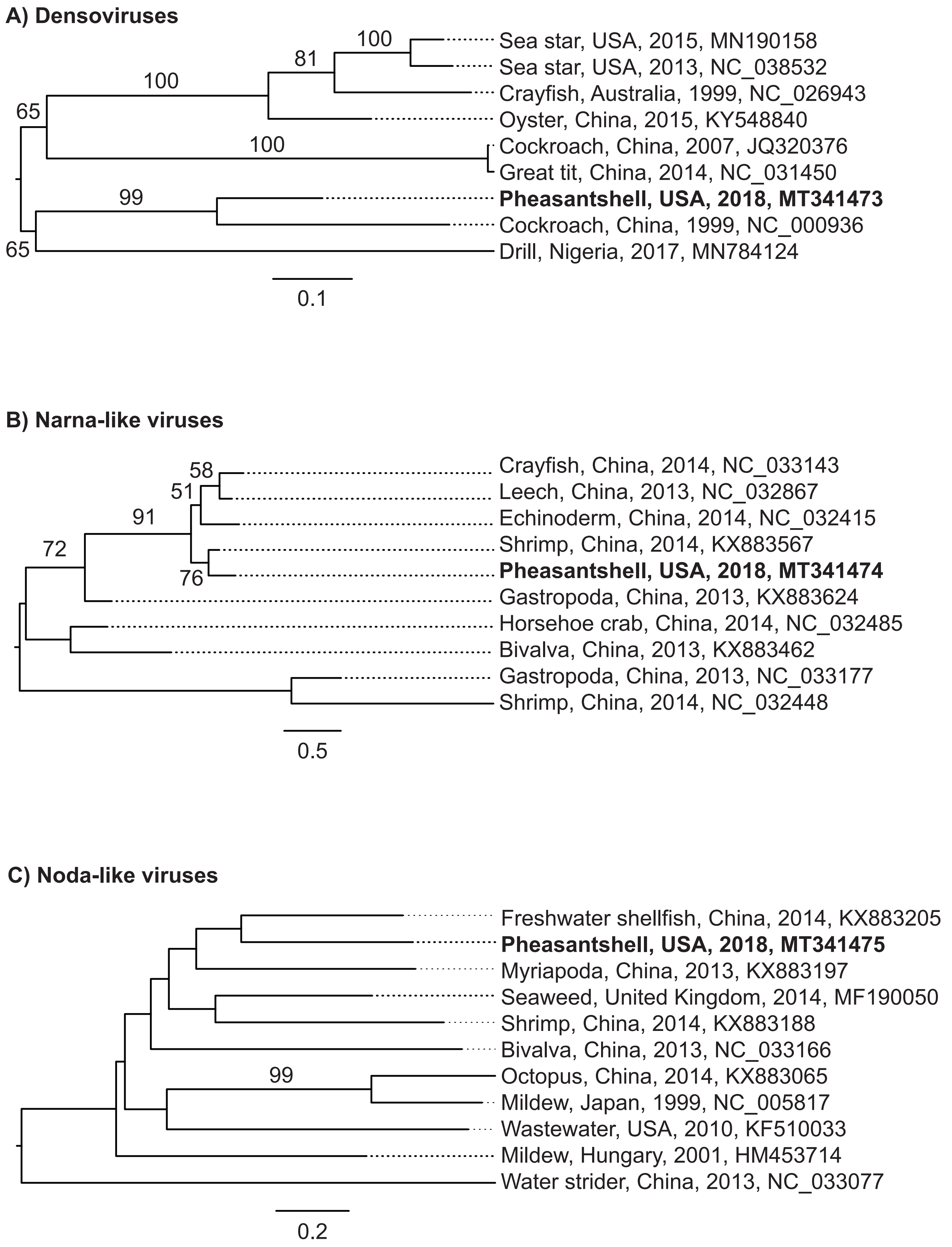
A) Densoviruses
100
81
100
65
100
99
65
Sea star, USA, 2015, MN190158
Sea star, USA, 2013, NC_038532
Crayfish, Australia, 1999, NC_026943
Oyster, China, 2015, KY548840
Cockroach, China, 2007, JQ320376
Great tit, China, 2014, NC_031450
Pheasantshell, USA, 2018, MT341473
Cockroach, China, 1999, NC_000936
Drill, Nigeria, 2017, MN784124
0.1
B) Narna-like viruses
58
51
91
72
76
Crayfish, China, 2014, NC_033143
Leech, China, 2013, NC_032867
Echinoderm, China, 2014, NC_032415
Shrimp, China, 2014, KX883567
Pheasantshell, USA, 2018, MT341474
Gastropoda, China, 2013, KX883624
Horsehoe crab, China, 2014, NC_032485
Bivalva, China, 2013, KX883462
Gastropoda, China, 2013, NC_033177
Shrimp, China, 2014, NC_032448
0.5
C) Noda-like viruses
99
Freshwater shellfish, China, 2014, KX883205
Pheasantshell, USA, 2018, MT341475
Myriapoda, China, 2013, KX883197
Seaweed, United Kingdom, 2014, MF190050
Shrimp, China, 2014, KX883188
Bivalva, China, 2013, NC_033166
Octopus, China, 2014, KX883065
Mildew, Japan, 1999, NC_005817
Wastewater, USA, 2010, KF510033
Mildew, Hungary, 2001, HM453714
Water strider, China, 2013, NC_033077
0.2

D) Picorna-like viruses (1)

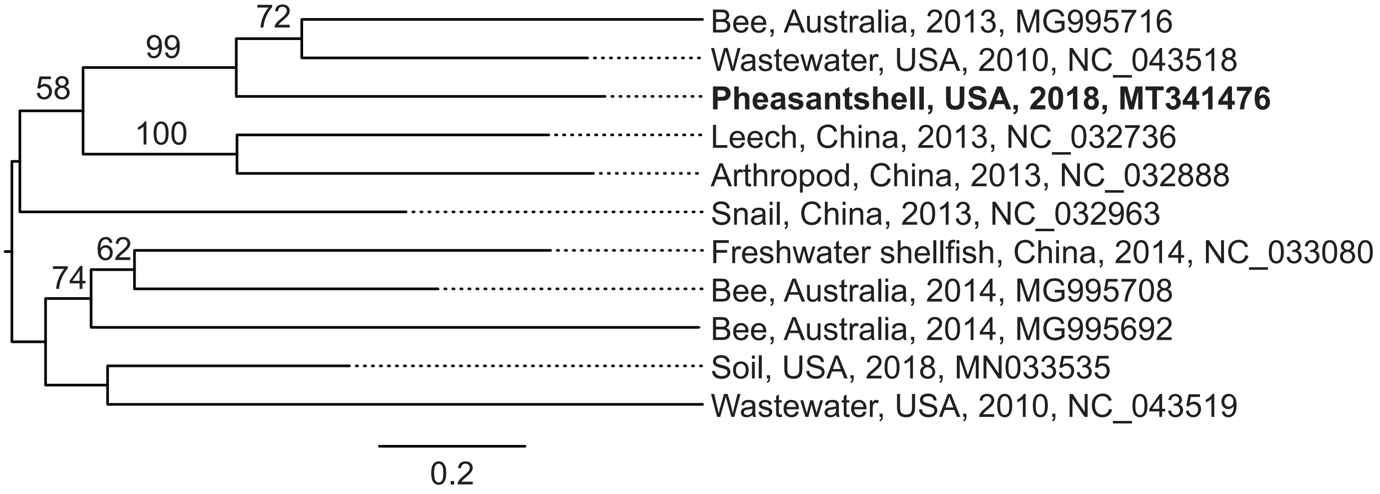

E) CRESS viruses

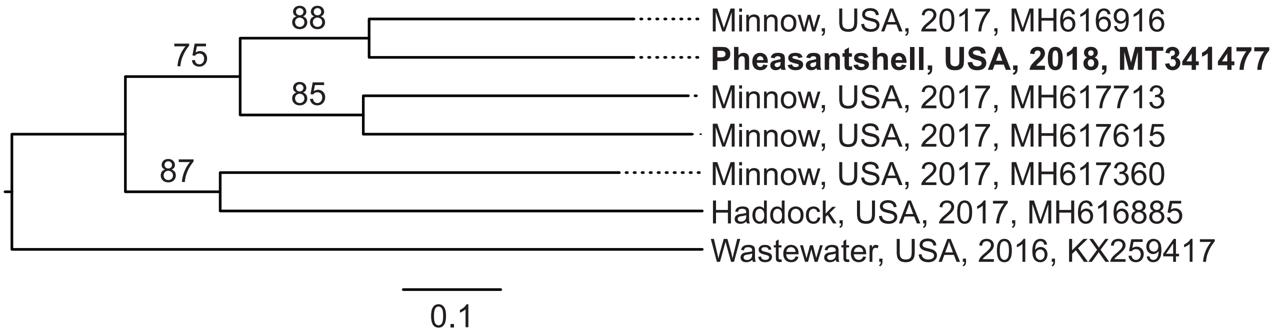

F) Picorna-like viruses (2)

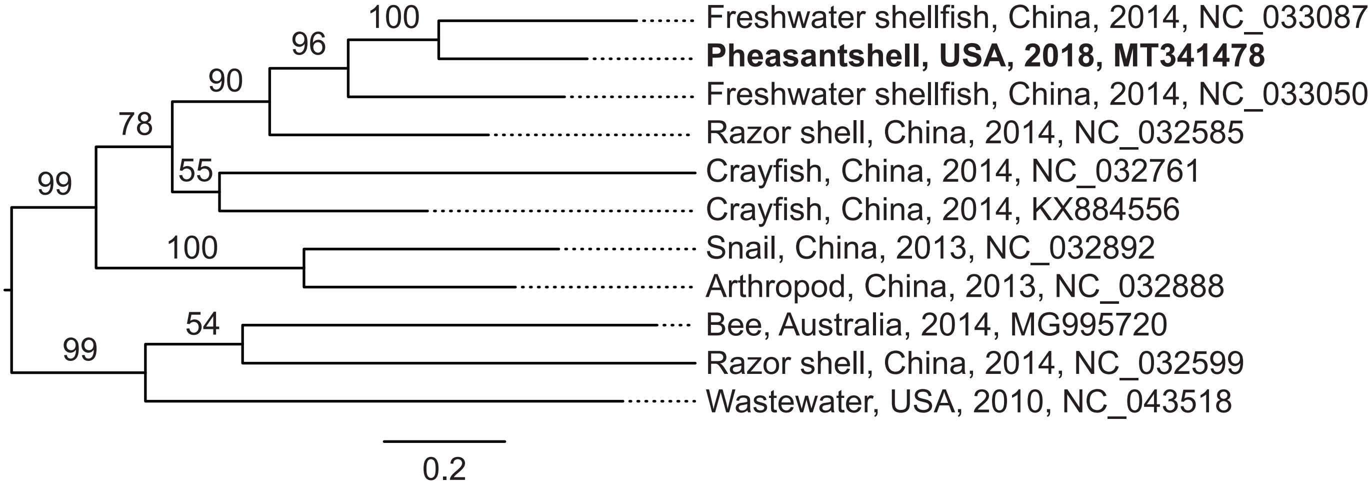

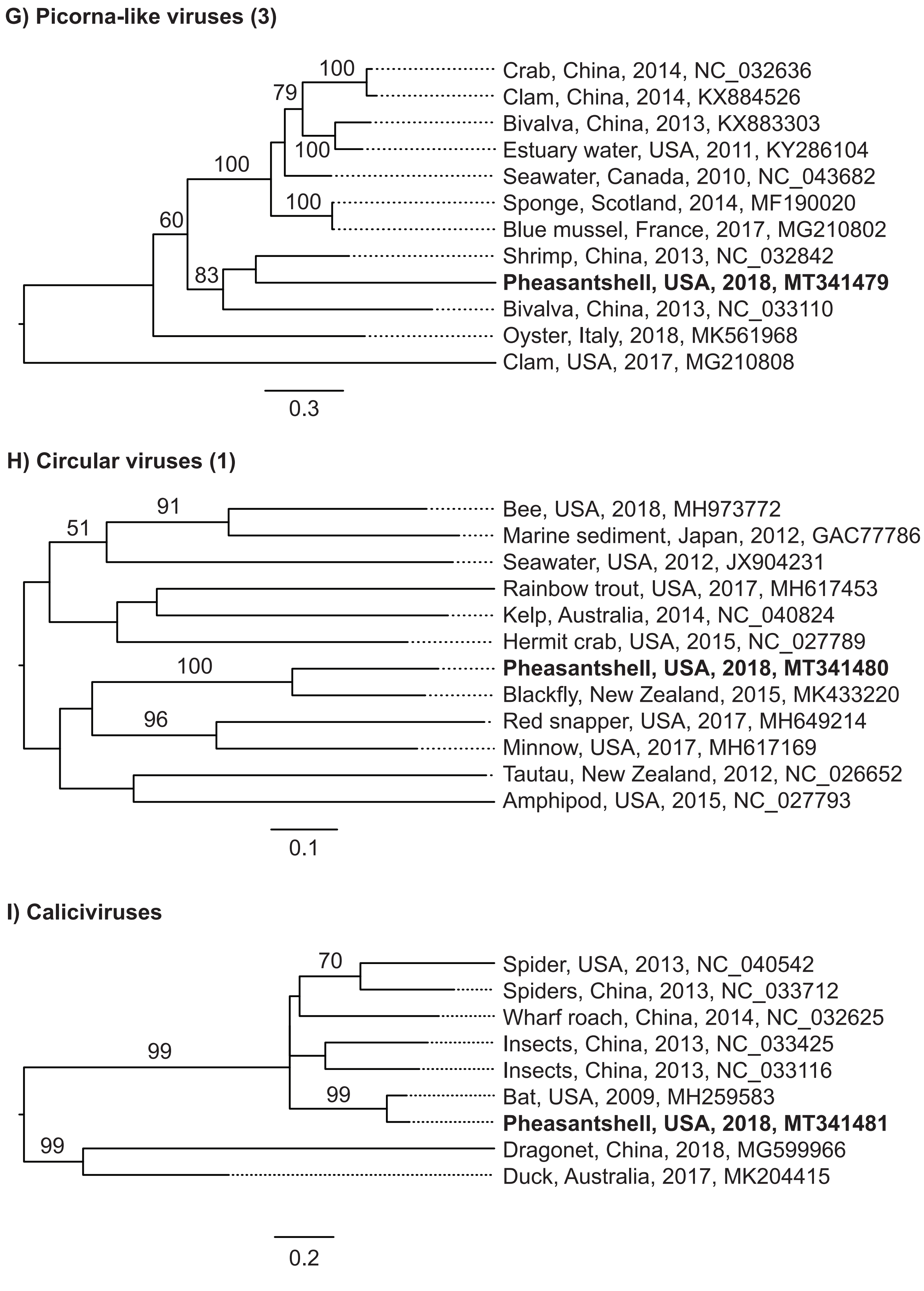

G) Picorna-like viruses (3)
100
79
100
100
60
100
83
Crab, China, 2014, NC_032636
Clam, China, 2014, KX884526
Bivalva, China, 2013, KX883303
Estuary water, USA, 2011, KY286104
Seawater, Canada, 2010, NC_043682
Sponge, Scotland, 2014, MF190020
Blue mussel, France, 2017, MG210802
Shrimp, China, 2013, NC_032842
Pheasantshell, USA, 2018, MT341479
Bivalva, China, 2013, NC_033110
Oyster, Italy, 2018, MK561968
Clam, USA, 2017, MG210808
0.3
H) Circular viruses (1)
91
51
100
96
Bee, USA, 2018, MH973772
Marine sediment, Japan, 2012, GAC77786
Seawater, USA, 2012, JX904231
Rainbow trout, USA, 2017, MH617453
Kelp, Australia, 2014, NC_040824
Hermit crab, USA, 2015, NC_027789
Pheasantshell, USA, 2018, MT341480
Blackfly, New Zealand, 2015, MK433220
Red snapper, USA, 2017, MH649214
Minnow, USA, 2017, MH617169
Tautau, New Zealand, 2012, NC_026652
Amphipod, USA, 2015, NC_027793
0.1
I) Caliciviruses
70
99
99
99
Spider, USA, 2013, NC_040542
Spiders, China, 2013, NC_033712
Wharf roach, China, 2014, NC_032625
Insects, China, 2013, NC_033425
Insects, China, 2013, NC_033116
Bat, USA, 2009, MH259583
Pheasantshell, USA, 2018, MT341481
Dragonet, China, 2018, MG599966
Duck, Australia, 2017, MK204415
0.2

J) Circular viruses (2)

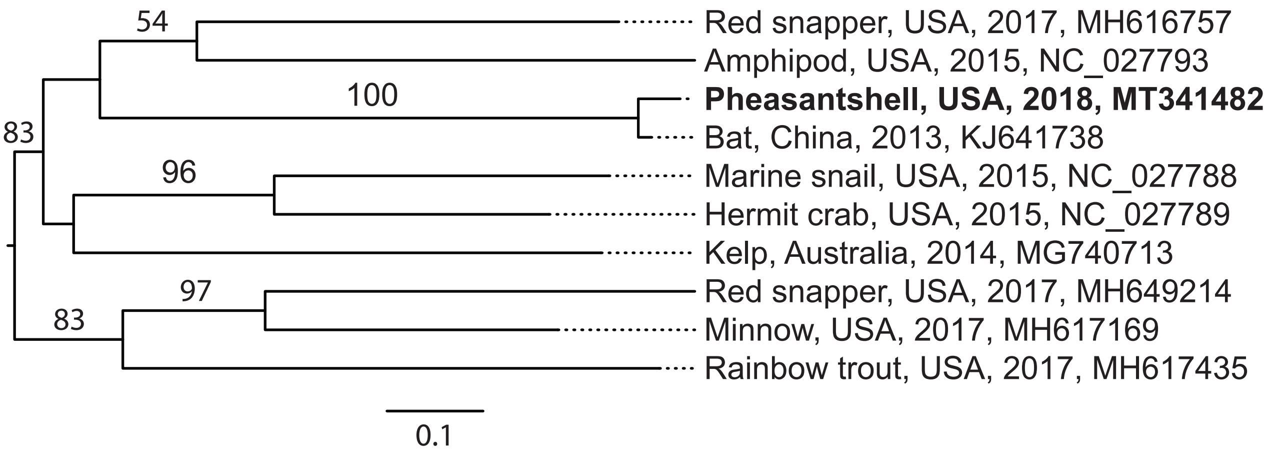

K) Dicistro-like viruses (1)

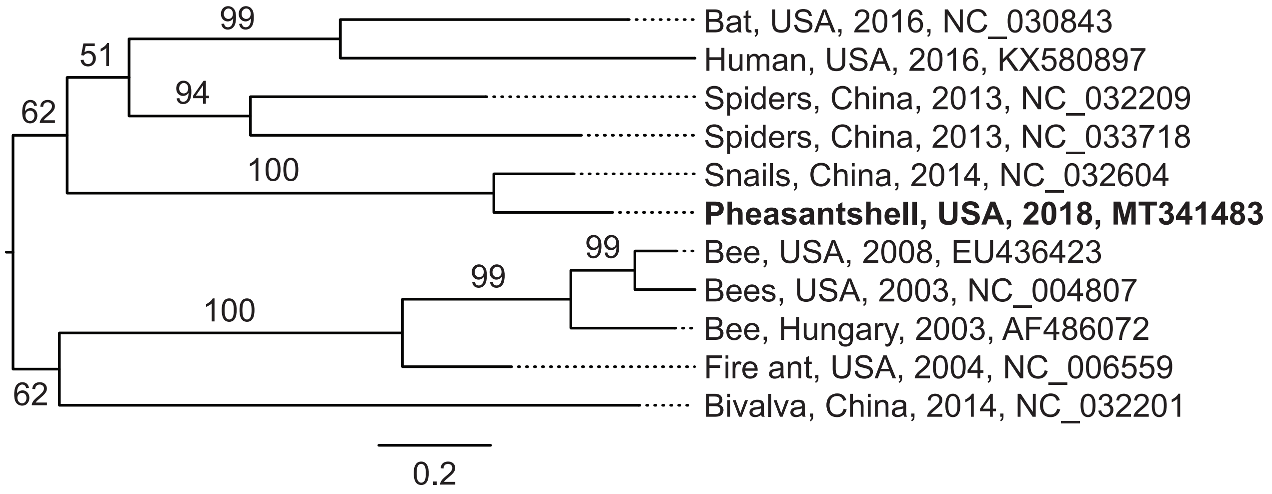

L) Tombus-like viruses

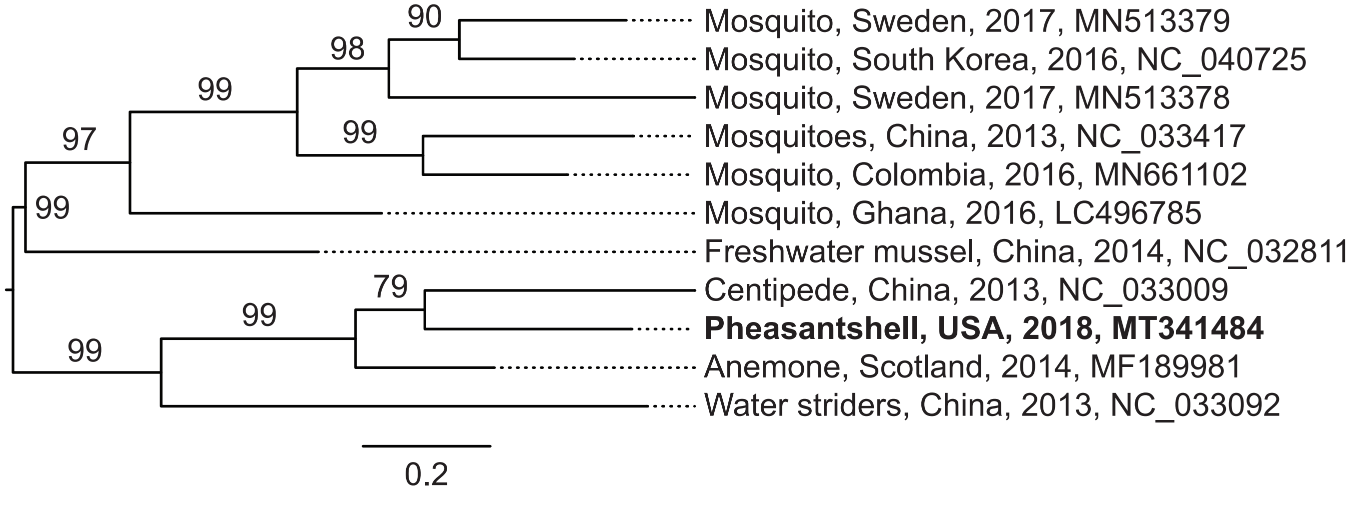

M) Sobemo-like viruses

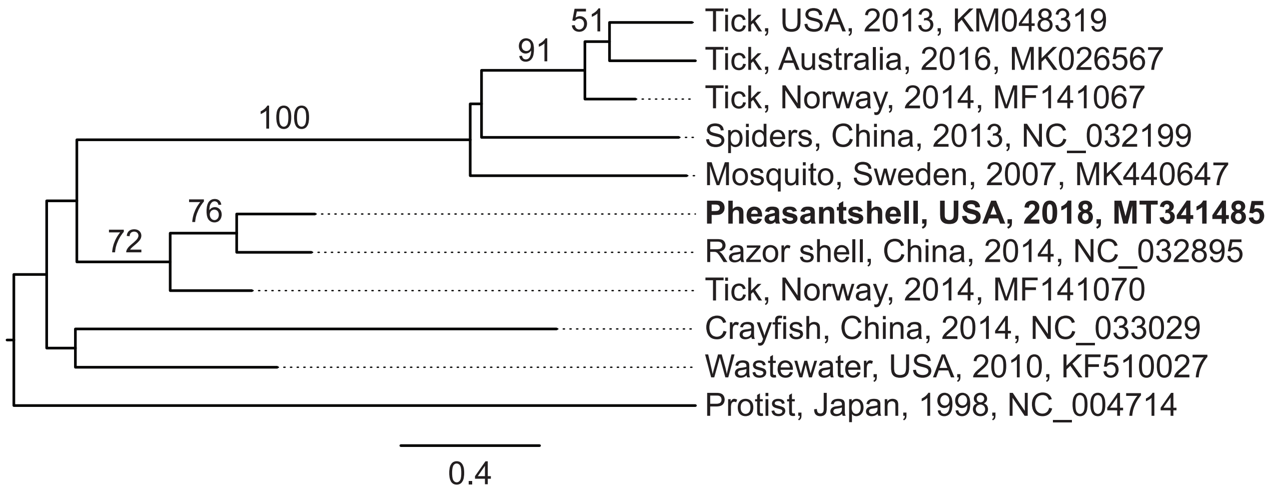

N) Dicistro-like viruses (2)

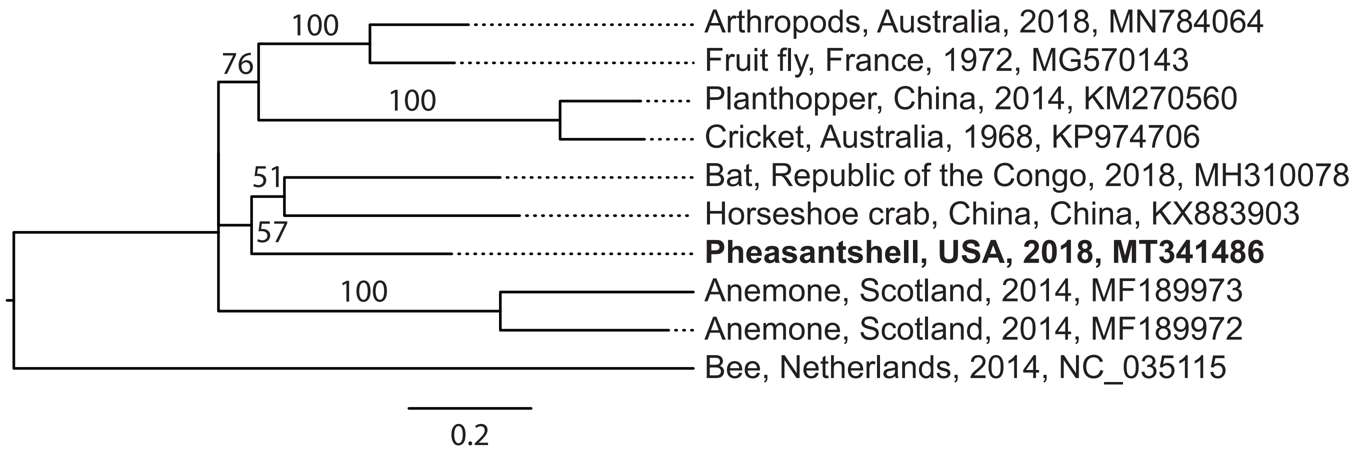

O) Picobirnaviruses

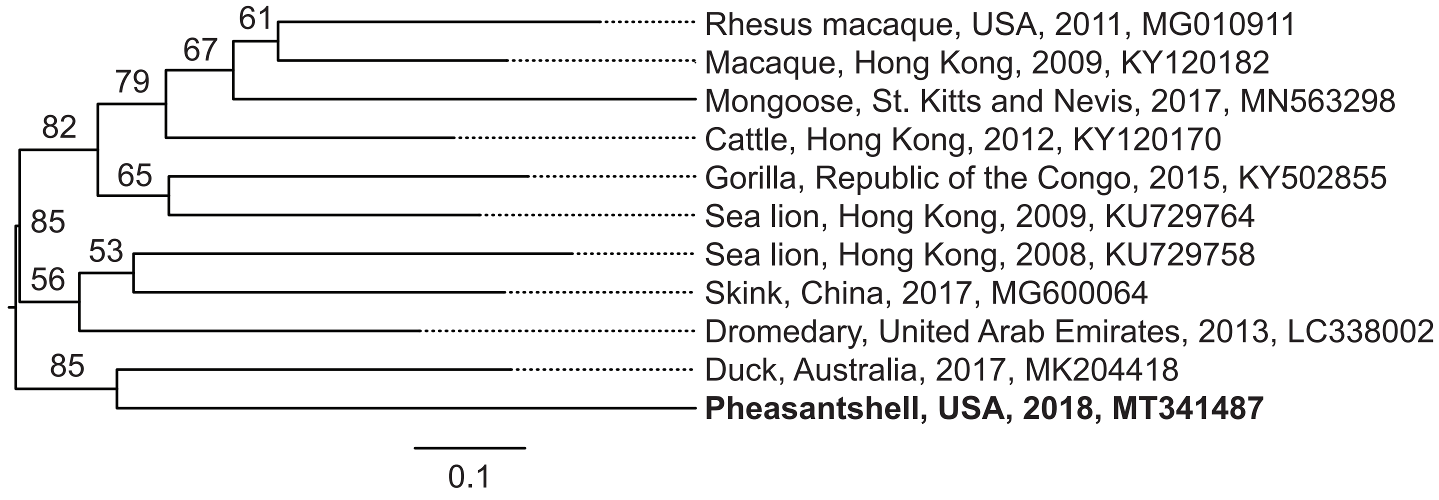

P) Picobirna-like viruses

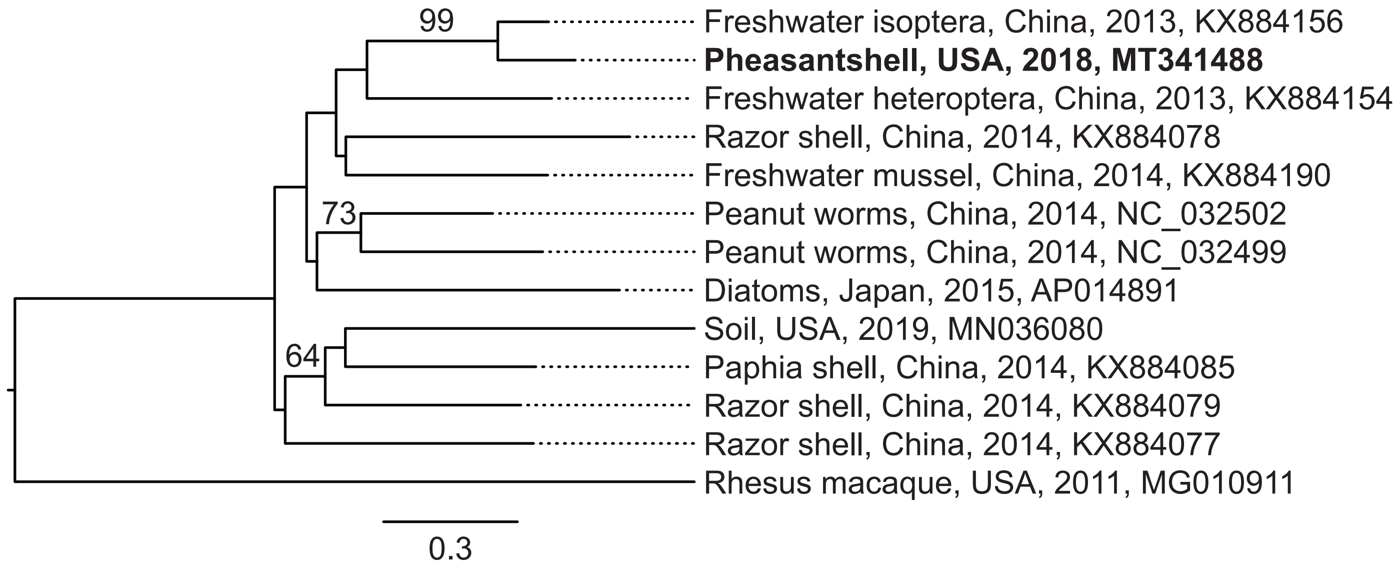

Q) Totiviruses

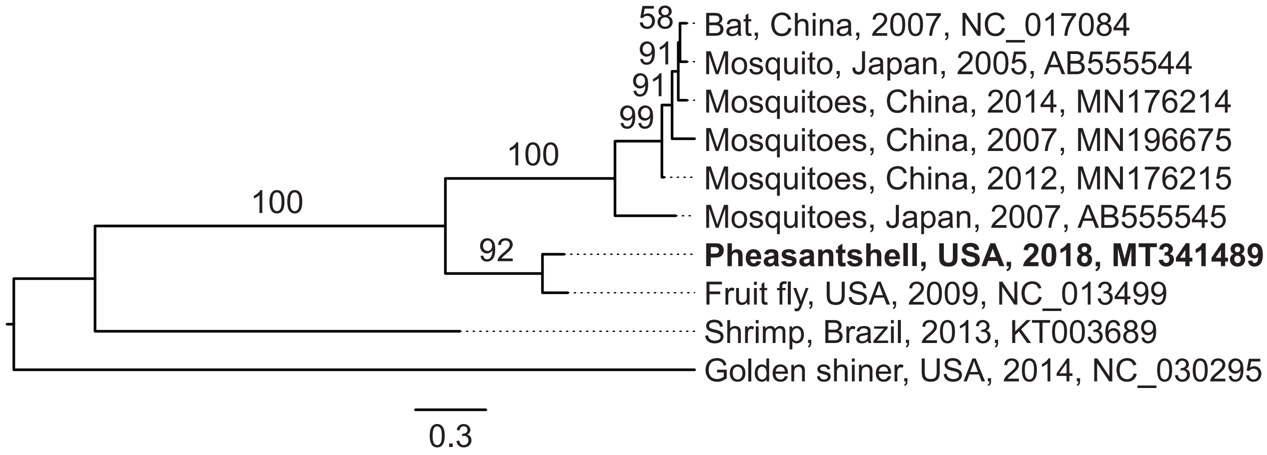

Table S1. Details of Clinch River pheasantshells (*Actinonaias pectorosa*) used in the analyses

Accession	Short ID	Status	Date	Location	Condition[1]	Length (mm)	Gravid[2]
AP102017-037	Y37	Case	10/20/17	Frost Ford		96.0	
AP102017-043	Y43	Case	10/20/17	Kyle's Ford		97.0	
AP102017-065	Y65	Case	10/20/17	Kyle's Ford		90.0	
AP102017-066	Y66	Case	10/20/17	Kyle's Ford		92.5	
AP110317-001	Z01	Case	11/3/17	Kyle's Ford		71.0	
AP110317-002	Z02	Case	11/3/17	Kyle's Ford		72.0	
AP110317-005	Z05	Case	11/3/17	Kyle's Ford		75.0	
AP110317-006	Z06	Case	11/3/17	Kyle's Ford		90.0	
AP110317-008	Z08	Case	11/3/17	Kyle's Ford		74.0	
AP110317-016	Z16	Case	11/3/17	Wallen's Bend		80.0	
AP110317-003	Z03	Case	11/3/17	Kyle's Ford		72.0	
20180925SIAPE-0007	B32	Case	9/25/18	Sycamore Island	4	95.0	Yes
20180925SIAPE-0011	B36	Case	9/25/18	Sycamore Island	5	69.0	
20180925SIAPE-0012	B37	Case	9/25/18	Sycamore Island	5	91.0	No
20180925SIAPE-0014	B39	Case	9/25/18	Sycamore Island	4	96.0	
20180925SIAPE-0015	B40	Case	9/25/18	Sycamore Island	5	101.0	Yes
20181024SFAPE-0001	C16	Case	10/24/18	Speers Ferry	4	103.0	No
20181025SIAPE-0012	C47	Case	10/25/18	Sycamore Island	4	78.0	No
20181025SIAPE-0013	C48	Case	10/25/18	Sycamore Island	4	86.0	Yes
20181025SIAPE-0021	C59	Case	10/25/18	Sycamore Island	4	97.0	No
20181025KFAPE-0007	C69	Case	10/25/18	Kyle's Ford	4	73.0	No
20181025KFAPE-0011	C73	Case	10/25/18	Kyle's Ford	4	70.0	No
20181025KFAPE-0012	C74	Case	10/25/18	Kyle's Ford	3	83.0	No
20181025KFAPE-0014	C76	Case	10/25/18	Kyle's Ford	3	88.0	Yes
20181025KFAPE-0015	C77	Case	10/25/18	Kyle's Ford	2	74.5	No
20181025KFAPE-0016	C78	Case	10/25/18	Kyle's Ford	3	94.0	Yes
AP102017-005	Y05	Control	10/20/17	Sycamore Island		104.0	
AP102017-015	Y15	Control	10/20/17	Wallen's Bend		84.0	
AP102017-027	Y27	Control	10/20/17	Frost Ford		89.0	
AP102017-044	Y44	Control	10/20/17	Kyle's Ford		85.5	
AP110317-009	Z09	Control	11/3/17	Kyle's Ford		94.0	
AP110317-010	Z10	Control	11/3/17	Kyle's Ford		61.0	
AP110317-013	Z13	Control	11/3/17	Wallen's Bend		61.0	
AP110317-015	Z15	Control	11/3/17	Wallen's Bend		84.0	
20180816SIAPE-0002	A10	Control	8/16/18	Sycamore Island	4	96.0	Yes
20180816SIAPE-0008	A16	Control	8/16/18	Sycamore Island	5	95.7	No
20180816WBAPE-0007	A23	Control	8/16/18	Wallen's Bend	4	91.6	No
20180816KFAPE-0002	A34	Control	8/16/18	Kyle's Ford		99.4	No
20180816KFAPE-0006	A38	Control	8/16/18	Kyle's Ford	5	83.8	No
20180925ARAPE-0004	B04	Control	9/25/18	Artrip	4	95.0	Yes
20180925SFAPE-0004	B17	Control	9/25/18	Speers Ferry	4	85.0	Yes
20180925SIAPE-0002	B26	Control	9/25/18	Sycamore Island	4	92.0	Yes
20180925SIAPE-0003	B27	Control	9/25/18	Sycamore Island	4	97.0	Yes
20180925SIAPE-0004	B28	Control	9/25/18	Sycamore Island	5	91.0	Yes
20180925SIAPE-0006	B31	Control	9/25/18	Sycamore Island	5	104.0	No
20180925WBAPE-0002	B44	Control	9/25/18	Wallen's Bend	4	84.4	No
20181025SIAPE-0002	C36	Control	9/25/18	Sycamore Island	5	93.0	No
20181025KFAPE-0001	C63	Control	9/25/18	Kyle's Ford	4	70.0	No
20181025KFAPE-0002	C64	Control	9/25/18	Kyle's Ford	3	88.0	Yes
20180925KFAPE-0020	B71	Control	9/26/18	Kyle's Ford	4	62.0	No
20180925KFAPE-0022	B73	Control	9/26/18	Kyle's Ford	3	78.0	No
20180925KFAPE-0024	B75	Control	9/26/18	Kyle's Ford	5	60.0	No
20181024ARAPE-0004	C04	Control	10/24/18	Artrip	4	106.0	Yes
20181024SFAPE-0013	C33	Control	10/24/18	Speers Ferry	5	82.0	No
20181025KFAPE-0004	C66	Control	10/25/18	Kyle's Ford	4	76.0	No
20181025KFAPE-0008	C70	Control	10/25/18	Kyle's Ford	3	58.0	No
20181025KFAPE-0009	C71	Control	10/25/18	Kyle's Ford	3	50.0	No
20181025KFAPE-0013	C75	Control	10/25/18	Kyle's Ford	3	88.0	Yes

[1]Body condition score was ranked on an ordinal scale of 1 (poor) to 5 (excellent). Blank cells represent missing data.

[2]Reproductive status (gravid or not gravid) was assessed during physical examintion. Bland cells represent missing data.

Table S2. Loads of 17 viruses in Clinch River pheasantshells (*Actinonaias pectorosa*)

		Virus[1]																
Short ID[2]	Status	A	B	C	D	E	F	G	H	I	J	K	L	M	N	O	P	Q
Y37	Case	0.83	0.00	0.00	0.00	0.00	0.00	0.00	0.00	0.00	0.00	0.00	0.00	0.00	0.00	0.00	0.00	0.00
Y43	Case	1.24	0.30	0.15	2.17	0.00	1.60	0.24	1.43	0.59	0.00	0.00	0.34	0.00	0.21	0.00	0.00	0.00
Y65	Case	0.48	0.00	0.00	1.52	0.00	0.42	0.00	0.00	0.00	0.98	0.00	0.00	0.00	0.00	0.00	0.00	0.00
Y66	Case	1.48	0.00	0.00	0.00	0.27	0.00	0.00	0.00	0.00	0.00	0.00	0.00	0.00	0.00	0.00	0.00	0.00
Z01	Case	0.18	0.00	0.00	0.00	0.00	0.00	0.00	0.00	0.00	0.00	0.00	0.00	0.00	0.00	0.00	0.00	0.00
Z02	Case	1.44	0.12	0.00	1.16	0.42	0.00	0.00	0.78	0.00	0.00	0.00	0.00	0.00	0.00	0.00	0.00	0.00
Z05	Case	1.93	0.00	0.00	0.00	0.00	0.00	0.00	0.40	0.00	0.00	0.00	0.00	0.00	0.00	0.00	0.00	0.00
Z06	Case	0.00	0.00	0.00	0.06	0.00	0.00	0.00	0.00	0.00	0.43	0.00	0.00	0.00	0.00	0.00	0.00	0.00
Z08	Case	0.00	0.00	0.00	0.06	0.41	0.28	0.00	0.00	0.00	0.93	0.00	0.00	0.00	0.00	0.00	0.00	0.00
Z16	Case	0.00	0.00	0.00	0.00	0.00	0.00	0.00	0.00	0.00	0.51	0.00	0.00	0.00	0.00	0.00	0.00	0.00
Z03	Case	0.00	0.00	0.00	1.51	0.00	0.11	0.00	0.00	0.38	0.00	0.00	0.00	0.00	0.00	0.00	0.00	0.00
B32	Case	0.00	0.04	0.09	0.37	0.31	0.00	0.00	0.00	0.05	0.15	0.00	0.00	0.00	0.00	0.00	0.00	0.00
B36	Case	1.67	0.00	0.00	0.11	2.13	0.00	0.00	0.00	0.00	0.20	0.10	0.00	0.00	0.00	0.00	0.00	0.00
B37	Case	0.95	0.51	0.00	2.54	0.67	0.98	0.00	0.00	1.17	1.00	0.00	0.00	0.00	0.00	0.00	0.00	0.00
B39	Case	1.32	0.00	0.00	0.66	1.54	0.00	0.00	0.00	0.00	1.08	0.00	0.00	0.00	0.00	0.00	0.00	0.00
B40	Case	0.00	0.00	0.00	1.46	0.00	0.00	0.00	0.00	0.00	1.48	0.00	0.00	0.00	0.00	0.00	0.00	0.00
C16	Case	0.00	0.00	0.00	0.00	0.00	0.00	0.00	0.00	0.00	0.00	0.00	0.00	0.00	0.00	0.00	0.00	0.00
C47	Case	3.09	2.56	1.30	2.39	1.08	0.64	1.60	0.97	1.45	1.13	1.17	1.26	0.89	1.51	0.00	1.47	1.73
C48	Case	0.72	0.00	0.00	1.07	1.20	0.94	0.00	0.65	0.14	2.29	0.00	0.09	0.00	0.00	0.00	0.00	0.00
C59	Case	0.35	0.00	0.00	0.00	1.40	0.00	0.00	0.39	0.00	1.16	0.00	0.00	0.00	0.00	0.00	0.00	0.00
C69	Case	0.55	0.00	0.00	0.11	0.79	0.18	0.13	0.77	0.00	1.24	0.00	0.00	0.00	0.00	1.05	0.00	0.00
C73	Case	0.19	0.00	0.00	0.00	0.10	0.00	0.00	0.00	0.00	0.21	0.00	0.00	0.00	0.00	0.00	0.00	0.00
C74	Case	0.95	0.00	0.00	0.00	0.81	0.00	0.00	0.62	0.00	0.97	0.00	0.00	0.00	0.00	0.00	0.00	0.00
C76	Case	0.00	0.00	0.00	0.13	0.84	0.52	0.00	0.62	0.00	1.30	0.00	0.00	0.00	0.00	0.00	0.00	0.00
C77	Case	0.32	0.00	0.00	0.00	1.71	0.00	0.00	0.00	0.00	0.92	0.00	0.00	0.00	0.00	0.00	0.00	0.00
C78	Case	1.36	0.07	0.00	0.57	0.00	0.00	0.00	0.00	0.00	0.75	0.00	0.00	0.00	0.00	0.00	0.00	0.00
Y05	Control	0.00	0.00	0.00	0.00	0.00	0.00	0.00	0.00	0.00	0.00	0.00	0.00	0.00	0.00	0.00	0.00	0.00
Y15	Control	0.00	0.00	0.00	0.00	0.00	0.00	0.00	0.47	0.33	0.00	0.00	0.00	0.00	0.00	0.00	0.00	0.00
Y27	Control	0.00	0.00	0.00	0.59	0.00	0.00	0.00	0.00	0.00	0.00	0.00	0.00	0.00	0.00	0.00	0.00	0.00
Y44	Control	0.00	0.00	0.00	0.08	0.49	0.00	0.00	0.00	0.00	0.36	0.00	0.00	0.00	0.00	0.00	0.00	0.00
Z09	Control	0.00	0.00	0.00	0.00	0.00	0.00	0.00	0.00	0.00	1.54	0.00	0.47	0.00	0.00	0.00	0.00	0.00
Z10	Control	0.32	0.00	0.00	0.53	0.00	0.16	0.00	0.00	0.00	0.68	0.00	0.00	0.00	0.00	0.00	0.00	0.00
Z13	Control	0.37	0.00	0.00	0.28	0.00	0.00	0.00	0.53	0.00	0.64	0.00	0.00	0.00	0.00	0.00	0.00	0.00
Z15	Control	0.56	0.00	0.00	0.58	0.20	0.00	0.00	0.00	0.00	0.32	0.00	0.00	0.00	0.00	0.00	0.00	0.00
A10	Control	0.00	0.00	0.00	0.00	0.00	0.27	0.00	0.00	0.00	0.32	0.00	0.00	0.00	0.00	0.00	0.00	0.00
A16	Control	0.19	0.00	0.00	0.00	0.00	0.00	0.00	0.29	0.00	0.96	0.00	0.00	0.00	0.00	0.00	0.00	0.00
A23	Control	0.44	0.00	0.00	0.15	0.00	0.00	0.00	0.00	0.00	0.90	0.00	0.00	0.00	0.00	0.00	0.00	0.00
A34	Control	0.14	0.00	0.00	0.04	0.97	0.12	0.00	0.00	0.00	0.47	0.00	0.00	0.10	0.00	0.00	0.00	0.00
A38	Control	0.00	0.07	0.00	0.00	0.00	0.07	0.00	0.00	0.00	0.24	0.00	0.00	0.07	0.00	0.00	0.00	0.00
B04	Control	0.73	0.00	0.00	0.19	0.00	0.00	0.00	0.17	0.00	1.15	0.00	0.00	0.08	0.00	0.00	0.00	0.00
B17	Control	0.00	0.00	0.00	0.00	0.20	0.00	0.00	0.00	0.00	0.00	0.00	0.00	0.00	0.00	0.00	0.00	0.00
B26	Control	0.00	0.00	0.00	0.08	0.40	0.44	0.00	0.00	0.24	1.47	0.00	0.00	0.00	0.00	0.00	0.00	0.00
B27	Control	0.00	0.00	0.00	0.08	0.50	0.21	0.00	0.00	0.42	1.33	0.00	0.00	0.12	0.00	0.00	0.00	0.00
B28	Control	0.00	0.00	0.00	0.30	0.33	0.00	0.00	0.00	0.00	2.13	0.00	0.00	0.00	0.00	0.00	0.00	0.00
B31	Control	0.00	0.00	0.00	0.09	0.51	0.00	0.00	0.00	0.00	0.85	0.00	0.00	0.00	0.00	0.00	0.00	0.00
B44	Control	0.00	0.00	0.00	0.51	1.57	0.00	0.00	0.00	0.26	0.00	0.00	0.00	0.00	0.00	0.00	0.00	0.00
C36	Control	0.54	0.00	0.00	0.00	0.00	0.00	0.00	0.00	0.00	0.86	0.00	0.00	0.00	0.00	0.00	0.00	0.00
C63	Control	0.00	0.00	0.00	0.06	0.15	0.00	0.00	0.00	0.00	0.80	0.00	0.00	0.00	0.00	0.00	0.00	0.00
C64	Control	0.00	0.00	0.00	0.14	1.10	0.00	0.00	1.33	0.00	2.08	0.00	0.00	0.00	0.00	0.00	0.00	0.00
B71	Control	0.00	0.00	0.00	0.00	0.00	0.00	0.00	0.00	0.00	0.78	0.00	0.00	0.00	0.00	0.00	0.00	0.00
B73	Control	0.00	0.00	0.00	0.00	0.00	0.00	0.00	0.00	0.00	0.89	0.00	0.00	0.00	0.00	0.00	0.00	0.00
B75	Control	0.41	0.00	0.00	0.12	0.80	0.00	0.00	0.00	0.00	0.00	0.00	0.00	0.00	0.00	0.00	0.00	0.00
C04	Control	0.39	0.00	0.00	0.04	0.00	0.00	0.00	0.00	0.14	1.36	0.00	0.00	0.00	0.00	0.00	0.00	0.00
C33	Control	0.00	0.00	0.00	0.00	0.24	0.62	0.00	0.00	0.19	1.63	0.00	0.00	0.00	0.00	0.00	0.00	0.00
C66	Control	0.27	0.00	0.00	0.13	0.81	0.54	0.00	0.76	0.00	0.60	0.00	0.00	0.00	0.00	0.00	0.00	0.00
C70	Control	0.00	0.00	0.00	0.44	0.38	0.00	0.00	0.00	0.00	0.00	0.00	0.00	0.00	0.00	0.00	0.00	0.00
C71	Control	0.00	0.00	0.00	0.06	0.48	0.17	0.00	0.00	0.28	0.00	0.00	0.00	0.00	0.00	0.00	0.00	0.00
C75	Control	0.00	0.00	0.00	0.00	0.12	0.00	0.00	0.00	0.17	0.34	0.00	0.00	0.00	0.00	0.00	0.00	0.00

[1]For details of viruses, see Table 1. Viral loads are presented as $\log_{10}$ viral reads per million total reads per kilobase of target sequence.

[2]See Table S1 for full details of Clinch River pheasantshells used in the analyses.
